# Hospital-acquired urinary tract infection point prevalence in Turkey: Differences in risk factors among patient groups

**DOI:** 10.1186/1476-0711-12-31

**Published:** 2013-11-04

**Authors:** Meltem Isikgoz Tasbakan, Raika Durusoy, Husnu Pullukcu, Oguz Resat Sipahi, Sercan Ulusoy

**Affiliations:** 1 Medical Faculty Department of Infectious Diseases and Clinical Microbiology, Ege University, Izmir, Turkey; 2 Medical Faculty Department of Public Health, Ege University, Izmir, Turkey

**Keywords:** Urinary tract infection, Nosocomial infections, *Escherichia coli*, Point prevalence, Epidemiology

## Abstract

**Background:**

The aim of this study was to determine the point prevalence of nosocomial urinary tract infections (UTIs) and to investigate risk factors for pathogen type (*E. coli* vs. others) and extended-spectrum beta-lactamase (ESBL) positivity among nosocomial UTI patients.

**Methods:**

A questionnaire consisting of 44 questions on demographic data and risk factors of UTI cases was sent to 51 tertiary care hospitals. Univariate and multivariate analyses were conducted.

**Results:**

The overall prevalence of UTI was 1.82% (483/26534). The prevalence of UTI was higher in intensive care units (ICUs) with 6.77% versus 1.45% outside ICUs. Hospitals of the Ministry of Health (compared to university hospitals), hospitals in less developed provinces and hospitals with bed capacity < 500 had higher UTI prevalence. Patients without a urinary catheter were more likely to have received immunosuppressive therapy, current corticosteroid use, renal transplantation and uterine prolapsus and less likely to have another infection outside the urinary tract, as compared to catheterized patients. Among the 422 culture-positive patients, the most common pathogen was *E. coli* (45.5%). The risk factors increasing the likelihood of *E. coli* in urine culture were being female, history of urinary tract operation, no use of antibiotics in the preceding three months and infection outside the urinary tract. There were 247 patients with *E. coli* or *Klebsiella spp.* positive in culture. Among these, 61% (n=151) were ESBL- positive. Among patients having *E. coli/Klebsiella* positive in culture, antibiotic use in the preceding three months and history of urinary tract operation were the independent risk factors significantly increasing the risk of ESBL.

**Conclusions:**

The reasons underlying the high prevalence of nosocomial UTIs, and a better understanding of the risk factors might lead to improved control of these infections.

## Introduction

Urinary tract infection (UTI) is the most commonly encountered hospital-acquired infection and the major risk factor is urinary catheterization [1]. According to reports from Turkey, 21-49% of hospital-acquired infections are urinary tract infections [2]. In a multi-center point prevalence study conducted in Turkey in 2001 where the same case definitions were used, the prevalence of hospital-acquired urinary tract infections had been reported as 1.7% among 13269 patients from 29 centers [3]. The importance of nosocomial infections has increased in the last decade and establishment of hospital infection committees and surveillance of nosocomial infections have become mandatory since 2005 for all the hospitals in Turkey [4]. In this multi-center point-prevalence study, it was aimed to determine the prevalence of hospital-acquired UTI among inpatients and to investigate risk factors determining pathogen type (*E. coli* vs. others) and extended-spectrum beta-lactamase (ESBL) positivity among nosocomial UTIs.

## Patients and methods

A questionnaire consisting of 44 questions was prepared to investigate demographic data (gender, age), risk factors, catheter use and characteristics of rooms of patients who had UTI (Additional file 1). The latest version of this form was given to two independent experts outside the study team for their expert views. The questionnaire reached its final version after a pilot study conducted in Ege University. The study was approved by the local ethics committee.

*Inclusion criteria:* Patients aged 18 years and above were included in the study. Cases having at least one of the symptoms like fever (> 38°C), pollakiuria, dysuria, suprapubic tenderness and having ≥ 10^5^ CFU/ml of one or two types of bacteria or culture-negative patients having at least two of the above-mentioned symptoms and one of the seven criteria defined by Centers for Disease Control and Prevention like nitrite test positivity, pyuria were included in the study [5]. All the cases were diagnosed by infectious diseases specialists. Asymptomatic patients were excluded.

*Study design:* A list of all public tertiary care hospitals in Turkey, i.e. University Hospitals and Training and Research Hospitals of the Ministry of Health, was obtained from the Ministry of Health Department of Inpatient Institutions’ statistical yearbook. Special branch hospitals like pediatric, ophthalmologic hospitals etc. were excluded. In total, 71 general tertiary care hospitals comprised the target group and 51 accepted to participate in the study (71.8% coverage; three rejected participation and the others did not respond or could not be contacted). The questionnaires and a data-entry spreadsheet were sent by e-mail to the participating specialists who formed the study group. The point-prevalence survey was conducted on 28 September 2011, based on all the nosocomial UTI cases who were present on the wards of the hospital on that day. All the wards in the participating hospitals were visited by the study group, questioning about patients with UTI symptoms. Patient data were collected by the detailed standard questionnaires, entered electronically to the spreadsheet and sent by each participating centre to the organizing centre via e-mail.

### Statistical analysis

Data were analyzed centrally. The total, intensive care unit (ICU) and non-ICU prevalence rates of each hospital was calculated using the number of UTI cases and the number of total, ICU and non-ICU beds occupied in that hospital on the survey date. The overall hospital-acquired UTI prevalence was calculated along with its 95% confidence interval (CI). Prevalences in ICUs and non-ICU wards were compared with paired samples t test. The provinces of the hospitals were classified according to the State Planning Organization’s 2011 developmental ranking [6]. The possible impacts of institution (Ministry of Health vs. university), bed capacity (< 500 vs. ≥ 500) and the province’s developmental level (the most developed 10 provinces vs. others) on the UTI prevalences of hospitals were analysed with Mann–Whitney U test, and the prevalence outside ICUs was compared according to institution using Student’s t test. Univariate analyses on the impacts of risk factors on the type of organism (*E. coli* vs. others) and the presence of ESBL among patients with *E. coli* or *Klebsiella spp*. in urine culture were conducted with the chi-square test. All the variables with a significant impact in univariate analyses were then entered into a multivariate logistic regression model. Odds ratios are presented with 95% confidence intervals. A p value less than 0.05 was considered as significant.

## Results

Data were collected from 51 tertiary care hospitals located in 29 out of 81 provinces of Turkey. The overall prevalence of UTI was 1.82% (n=483, 95% CI 1.819-1.822) according to our survey conducted in 51 hospitals comprising 26534 occupied beds in total, with a range between 0.00-5.26% among individual hospitals. The prevalence of UTI in ICUs was 6.77% (n=127; 95% CI 6.759-6.782) with the range 0.00-32.26% among different hospitals and 1.45% (n=356; 95% CI 1.445-1.448) outside ICUs, with a range of 0.00-4.90% among different hospitals.

Overall, ICU and non-ICU UTI prevalence in hospitals in less developed provinces and hospitals with < 500 bed capacities was significantly higher. Hospitals of the Ministry of Health had a higher overall prevalence of UTI as compared to University Hospitals, while their ICU prevalence was much higher but non-ICU prevalence significantly lower than university hospitals (Table 1). Two hospitals did not have any UTI cases. The mean number of nosocomial UTI per hospital was 9.5 ± 7.4 (range 0–34; median 7), with a total of 483 UTI cases on the survey date.

**Table 1:** UTI prevalence among different hospital types and units

**Categories**	**Number of hospitals**	**Overall UTI prevalences (95% confidence intervals)**					
		**Total no. of patients / Total no. beds**	**Overall prevalence**	**ICU patients**	**ICU prevalence**	**Non-ICU patients**	**Non-ICU prevalence**
Institution							
Ministry	15	143 / 7526	1.900 (1.897-1.903)	46 / 559	8.229 (8.206-8.252)	97 / 6967	1.392 (1.390-1.395)
University	36	340 / 19008	1.789 (1.787-1.791)	81 / 1302	6.221 (6.208-6.234)	259 / 17706	1.463 (1.461-1.465)
Provincial development							
First 10 provinces	27	292 / 16308	1.791 (1.789-1.793)	75 / 1127	6.655 (6.640-6.669)	217 / 15181	1.429 (1.428-1.431)
Remaining provinces	24	191 / 10226	1.868 (1.865-1.870)	52 / 734	7.085 (7.066-7.103)	139 / 9492	1.464 (1.462-1.467)
Bed capacity							
< 500 beds	28	185 / 8743	2.116 (2.113-2.119)	51 / 743	6.864 (6.846-6.882)	134 / 8000	1.675 (1.672-1.678)
≥ 500 beds	23	298 / 17791	1.675 (1.673-1.677)	76 / 1118	6.798 (6.783-6.813)	222 / 16673	1.332 (1.330-1.333)

The mean age of the patients was 59.1 ± 18.3 (range 18–96), with 53.6% (n=260) ≥ 60 years and 51.3% (n=248) female. Among these, 63.4% (n=306) were hospitalized in non-surgical (medical) departments vs. 36.6% (n=177) in surgical departments and totally 26.1% (n=126) were in the ICUs of the departments. Except 12 patients, all patients had at least one risk factor for UTI. The prevalence of each risk factor surveyed is shown in Table 2, stratified and compared according to the catheter status.

**Table 2:** Predisposing factors for urinary tract infections (UTIs) * (n=483)

	**Urinary catheter**		**Total**
Risk factor	Present (309) n (%)	Absent (174) n (%)	n (%)
Antibiotic use in preceding 3 month	220 (71.2)	118 (67.8)	338 (70.4)
UTI in the preceding year	127 (41.1)	80 (46.0)	207 (43.3)
Infection outside the urinary tract	131 (42.4)	35 (20.1)	166 (34.4)
Diabetes mellitus	80 (25.9)	51 (29.3)	131 (27.1)
Chronic renal failure	65 (21.0)	37 (21.3)	102 (21.2)
Immunosuppressive therapy in preceding 6 month	43 (13.9)	46 (26.4)	89 (18.4)
Urinary tract operation in preceding 6 month	55 (17.8)	33 (19.0)	88 (18.3)
Prostate hypertrophy	49 (15.9)	32 (18.4)	81 (18.6)
Current corticosteroid use	41 (13.3)	35 (20.1)	76 (15.8)
Urinary tract anomaly	39 (12.6)	19 (10.9)	58 (12.0)
Urethral stent	17 (5.5)	6 (3.4)	23 (4.8)
Urinary reflux	9 (2.9)	8 (4.6)	17 (3.5)
Renal transplantation	3 (1.0)	14 (8.0)	17 (3.5)
Uterine prolapsus	3 (1.0)	8 (4.6)	11 (4.4)

*Some patients had more than one predisposing factor.

Among the UTI cases, 309 had a urinary catheter. Catheterization was performed by a doctor in 261 patients (85%), by a nurse in 32 (10%), undergraduate intern doctor in 5 (2%), the patient himself in 6 (2%), and the catheterization team in one patient (0.3%). Among patients having a urinary catheter, only one was on day 0, four were on the 1^st^ day and 17 on the 2^nd^ day of their catheter. The distribution of patients according to the length of time the catheter remained in situ is shown in Table 3 along with other characteristics of catheterized patients.

**Table 3:** Characteristics of patients having a urinary catheter (n=309)

**Characteristic**	**n (%)**
Catheterization required	288 (93.2)
**Length of time the catheter remained in situ (day)**	
0-2	22 (7.1)
3-7	87 (28.2)
8-29	143 (46.3)
30 and over	57 (18.4)
**The place where catheterization was performed**	
Ward	163 (52.7)
ICU	90 (29.4)
Emergency ward	43 (14.1)
Operating room	11 (3.6)
Outpatient clinic	2 (0.7)
Drainage bag under the bed’s level	295 (96.0)
Drainage bag in proper position	256 (83.4)
Drainage bag with outlet tap	253 (82.4)
Catheter set present	201 (65.5)
Routine catheter change applied	106 (34.5)
Drainage bag touching the floor	45 (14.6)

Among the patients, 24.0% were staying in single rooms, 24.4% in double rooms and the remaining in rooms with three or more patient beds. A hand disinfectant was present in 77.0% of the patient rooms and 45.6% had a private toilet in the room.

Among the 483 patients with UTI, urine culture specimens were tested for 477 patients, of whom 467 had complete information on the urine culture results, 9.6% (n=45) being negative. Among the 422 culture-positive patients, the five leading microorganisms were *E. coli* 45.5% (n=192), *Candida spp.* 15.9% (n=67), *Klebsiella spp.* 13.3% (n=56), *Enterococcus spp.* 10.2% (n=43) and *Pseudomonas* spp. 10.0% (n=42). Of culture-positive patients, 4.9% (n=21) had mixed infection with two microorganisms simultaneously in the urine culture.

We explored the impact of UTI and other risk factors on the presence of *E. coli* –the leading microorganism in the study group with 45.5%– vs. other microorganisms in urine or blood culture. Significant risk factors are shown in Table 4. The mean age of patients with *E. coli* and patients with other microorganisms did not differ significantly. Other non-significant risk factors were: type of ward (internal vs. surgical), UTI in the preceding year, current corticosteroid use, immunosuppressive therapy in the preceding 6 months, diabetes mellitus, chronic renal failure, renal transplantation, prostate hypertrophy, urinary reflux and urethral stent.

**Table 4:** **The impact of UTI and other risk factors on the presence of *E. coli *** **vs. other microorganisms in culture (n=425)**

**Risk factor**	**Categories**	**n**	***E. coli *** **+ n (%)**	**Univariate p**	**Multivariate OR (95% CI)**
Gender	Male	209	86 (41.1)	0.067	0.63 (0.40-0.97)*
	Female	216	108 (50.0)		1
Clinic	Ward	304	160 (52.6)	< 0.001	0.64 (0.34-1.20)
	ICU	120	33 (27.5)		1
Toilet in patient’s room	Yes	188	99 (52.7)	0.010	1.15 (0.72-1.86)
	No	232	93 (40.1)		1
Hand disinfectant in patient’s room	Yes	334	142 (42.5)	0.005	1
	No	86	51 (59.3)		1.33 (0.78-2.26)
UTI RISK FACTORS					
Antibiotic use in preceding 3 mo.	Yes	312	129 (41.3)	0.002	0.56 (0.35-0.90)*
	No	111	65 (58.6)		1
Urinary tract operation in preceding 6 mo.	Yes	72	43 (59.7)	0.009	1.90 (1.06-3.40)*
	No	353	151 (42.8)		1
Urinary tract anomaly	Yes	47	27 (57.4)	0.082	1.26 (0.64-2.51)
	No	377	166 (44.0)		1
Infection outside the urinary tract	Yes	159	50 (31.4)	< 0.001	1
	No	266	144 (54.1)		1.74 (1.08-2.80)*
Catheter in the urinary tract	Yes	276	105 (38.0)	< 0.001	1
	No	148	89 (60.1)		1.54 (0.95-2.49)

*p< 0.00.

There were 247 patients with *E. coli* or *Klebsiella spp.* positive in culture. Among these, 61% (n=151) were ESBL-positive. The associations between known UTI risk factors on ESBL were explored and the significant associations are presented in Table 5. There was no difference in the mean age of ESBL positive and negative patients (58.0 ± 18.4 and 59.4 ± 19.0 years, respectively, p=0.590). Type of ward (medical vs. surgical), clinic (ward vs. ICU), presence of toilet in patient’s room, hand disinfectant in patient’s room, number of patient beds in the room, UTI in the preceding year, current corticosteroid use, immunosuppressive therapy in the preceding 6 months, diabetes mellitus, chronic renal failure, renal transplantation, urinary tract anomaly, prostate hypertrophy, urinary reflux, infection outside the urinary tract, catheter in the urinary tract and urethral stent did not have a significant impact on the presence of ESBL among patients having *E. coli/Klebsiella spp.* positive in culture.

**Table 5:** **The impact of UTI and other risk factors on the presence of ESBL among patients having *E. coli/Klebsiella spp. *** positive in culture (n=247**)

**Risk factor**	**Categories**	**n**	**ESBL+ n (%)**	**Univariate p**	**Multivariate OR (95% CI)**
Gender	Male	118**	80 (67.8)	0.032	1.12 (0.69-1.82)
	Female	130**	71 (54.6)		1
Antibiotic use in preceding 3 mo.	Yes	170	115 (67.6)	0.004	2.24 (1.34-3.74)*
	No	75	36 (48.0)		1
Urinary tract operation in preceding 6 mo.	Yes	51	40 (78.4)	0.005	2.17 (1.15-4.11)*
	No	195	111 (56.9)		1

*p< 0.005 *One case had mixed infection with *E. coli* and *Klebsiella spp.*

Blood culture was performed in 308 of the cases, of which 101 (32.8%) yielded an etiologic agent. In 38 patients, the same microorganism was positive in both urine and blood culture. The five leading micro-organisms found in blood cultures were *E. coli* (22), *Candida spp* (18), *Klebsiella spp* (12), *Acinetobacter spp* (12) and *S. aureus* (10).

In 165 patients, the presence of a simultaneous infection at another site was determined. Among these, 78 patients had pneumonia, 26 had soft tissue infection, 25 bacteremia, nine had surgical site infection, six gastroenteritis, five candidemia, four infective endocarditis, three septicaemia, two meningitis and one had herpes keratitis. Six patients had multiple infectious foci.

## Discussion

Determination of their prevalence and risk factors are keys to the prevention of hospital acquired urinary tract infections. The first point prevalence study carried out in 2001 in Turkey had found UTI prevalence as 1.7% [3]. Our present study, which was conducted ten years later, has included more than twice hospitals and patients and has found a prevalence of 1.82%. Sartor et al. had found a decrease in UTI prevalence from 1.8% to 1.1% in the point prevalence study they had repeated five years apart [7]. Askarian et al. have reported a UTI prevalence of 1.4% in their one-day point prevalence study they had repeated in four different days [8]. A decrease in the hospital acquired UTI prevalence could have been anticipated in Turkey, due to the recent legal implications. Considering the shortage of physicians, nurses and other health staff due to economic constraints, the stability of the UTI prevalence instead of an increase could be considered as the success of the infection control legislation. However, much more efforts are needed to reach the target of zero infection rates.

The prevalence of UTI is higher in ICUs. A study from Scotland has also found a significantly higher prevalence in ICUs (27.1% vs 9.3%) [9]. The most common reason of this higher prevalence is the application of catheters [10]. There is especially a link between the length of time the catheter remained in situ and the development of infection [11]. In our study, the very low number of patients on day 0–2 of the catheter (7.2%) and the high majority of patients on ≥ 3 days (92.8%) is in concordance with this well established link.

The total ICU and non-ICU prevalence rates were affected by hospital type, developmental level of the province and hospital size. While no significant difference was found according to the number of beds in the first point prevalence study conducted in Turkey in 2001, the point prevalence in hospitals with < 200 beds was much higher than the larger hospitals (2.8% vs. 1.4-1.7%) [3]. Our study has found a significantly higher prevalence in hospitals with < 500 beds as compared to hospitals with 500 beds and over. This might be related to less severe hospital infection control protocols in smaller-scale hospitals. As for type of institution, the lower overall prevalence found in university hospitals might be due to better infection control measures, especially in ICUs, but the higher prevalence in non-ICU wards might be attributed to the hospitalization of more complicated patients in university hospitals. The significantly lower prevalence in the most developed 10 provinces of Turkey might be due to more favorable economic conditions of the hospitals.

The most common risk factors identified in our study were the use of antibiotics in the preceding three months, urinary catheter, UTI in the preceding year and diabetes, in concordance with previous studies [12].

*Enterobacteriaceae* includes the most common pathogens associated with UTI. Other predominant pathogens are *Candida* species, *Enterococci* and *P. aeruginosa*. In another multi-national (including Turkey) and multi-center study, a total of 1762 isolates were collected from 38 centers in 11 countries from patients with UTIs in 2009 and 2010. *Enterobacteriaceae* comprised 86.0% of the isolates, of which *E. coli* (56.5%) and *K. pneumoniae* (13.8%) were the two most common species [13]. The most common pathogen in our study was *E. coli* as well, with 45.5%. The risk factors increasing the likelihood of *E. coli* in urine culture were female gender and urinary tract operation in the preceding six months. Female gender is an already established risk factor for community-acquired UTIs. Male gender, antibiotic use in the preceding three months and having an infection outside the urinary tract were significant risk factors increasing the likelihood of contracting other microorganisms than *E. coli* in the culture. Some other risk factors that were significant in univariate analyses lost significance in multivariate analyses. To our knowledge, this study is the first exploring risk factors that determine the pathogen type in UTIs.

*Candida* spp*.* was the second most common pathogen in our study with 15.9%. Yeasts that were the third most common pathogens in the point prevalence study conducted 10 years ago have risen to the second position. Many studies indicate that at least 10%–15% of hospital acquired UTIs are caused by *Candida* species. Candiduria is especially common in ICUs and may represent the most frequent UTIs encountered in adult surgical ICUs [14]. In a multi-center study conducted in ICUs in Turkey, the most common pathogen in catheter-associated UTIs were *Candida* species with 44.9% [15]. The fact that our patients had a high rate of antibiotic use and the high prevalence of ≥ 3 days urinary catheterization might have affected this outcome. The rise in the proportion of yeasts in catheterized patients merits special attention.

Antimicrobial resistance is increasing in bacteria isolated from both nosocomial and community-acquired UTIs [3]. ESBL-producing *E. coli* and *K. pneumoniae* are increasingly becoming of concern in many parts of the world [16]. The rise of ESBL positivity observed in Turkey has also been shown in this study [17]. Antibiotic use in the preceding 3 months and urinary tract operation in the preceding 6 months were significant risk factors for ESBL among patients positive for *E. coli* or *Klebsiella spp*, both of them being already established significant risk factors in the literature [18]. The increased risk in male gender in univariate analyses disappeared after adjustment, which might show bias due to the increased risk of urinary tract operations compared to women in univariate analyses.

Since 2006, when the national hospital infection surveillance system was established and surveillance became mandatory, all of the hospitals in Turkey have been reporting their hospital infection rates to the Ministry of Health. The government and Turkish Society of Hospital Infections and Control started quality control studies and educational programs in all regions of Turkey [19]. These relatively low rates of UTIs which are comparable with previous studies carried out in Iran and France can also be the result of educational and infection control programs [7,8].

Hand washing is a very important and cheap method for improving the hospital infection rates. Although there are not data related to the all study centers, the published hand washing studies from some of the study centers (Table 6) report a compliance rate of maximum 40%. Hence, hand washing compliance is poor and needs to be increased in most parts of Turkey [20-25].

**Table 6:** Published hand washing data in the study centers

**% (n)**	**Setting**	**Referances**
12.9 (298)	İstanbul University	20
40 (298)	Hacettepe University	21
20.8 (487)	Akdeniz University	22
5.3 (1286)	Ege University	23
23.04 (204)	Sakarya Training and Research Hospital (2010)	24
36.7 (364)	Sakarya Training and Research Hospital (2011)	24
28.9 (819)	Ankara Numune Training and Research Hospital	25

(n: Hand washing observation).

The most important limitation of this study is the lack of a control group, which could have enabled a clearer understanding of risk factors of hospital acquired UTI. Another limitation is the one-day duration of the study, which enabled only the recording of the ESBL ratio of the microorganisms found in urine cultures to the questionnaire.

In conclusion, the reasons underlying the high prevalence of nosocomial UTIs, which has not ameliorated in 10 years’ time despite the new legislation and its application should be investigated. A better understanding of the risk factors might lead to improved control of these infections.

## Competing interests

The authors declare that they have no competing interests.

## Author’s contributions

MIT conceived of the study, coordinated the data collection and drafted the manuscript. RD participated in its design, performed the statistical analysis and drafted the manuscript. HP helped the coordination of data collection. ORS drafted the manuscript and interpreted data. SU coordinated the study and data collection. The study group has collected the data. All authors and study group read and approved the final manuscript.

## Supplementary Material

Additional file 1 CASE REPORT FORM. Click here for file
