# Supplementary material for: Hospital-acquired urinary tract infection point prevalence in Turkey: Differences in risk factors among patient groups
Source: Ann Clin Microbiol Antimicrob. 2013 Nov 4;12:31. doi: 10.1186/1476-0711-12-31 (PMC4228335; doi:10.1186/1476-0711-12-31)
Supplement: Additional file 1 — CASE REPORT FORM. [file 1476-0711-12-31-S1.doc]

**CASE REPORT FORM**

**The prevalance of Hospital Acquired Urinary System Infections in Turkey:**

**Multi-center Point Prevalance Study**

**Case Number:**

1. Name- Surname: …………………. 2.Gender: 🞎 F 🞎 M 3.Birth Date: …………..

4. Date of Admission to the Hospital: ….. / ….. / 201....

5. Clinic: ……………………………..

6. Date of Admission to the ICU (if the patient is in ICU) : ….. / ….. / 201....

**Risk factors of Urinary Tract Infection *(please evaluate all of the risk factors below and tick off them)***

|  | Present | Absent |
| --- | --- | --- |
| 7. Antibiotic usage in the previous 3 months |  |  |
| 8. Name of the antibiotic ( If used) |  |  |
| 9. Urinary Tract Infection within the last year |  |  |
| 10. Urologic surgical intervention within last 6 months |  |  |
| 11. Corticosteroid usage |  |  |
| 12. Immunsupressive treatment |  |  |
| 13. Diabetes mellitus |  |  |
| 14. Chronic Renal Failure |  |  |
| 15. Renal Transplantation |  |  |
| 16. Urinary System Anomaly (name of the anomaly) |  |  |
| 17. Uterine Prolapsus |  |  |
| 18. Benign Prostatic Hyperplasia |  |  |
| 19. Vesicoureteral Reflux |  |  |
| 20. Infection at other sides of body |  |  |
| 21. If it exists : 🞎 Pneumonia 🞎 Surgical infection 🞎 Other: ……………………………… | | |
| 22. Urinary System Catheter |  |  |
| 23. Urethral Stent |  |  |

24.Urinary culture was performed from by? 🞎 Catheter 🞎 Mid urine voiding

25.Microorganism cultured from urinary culture : ……………………………………………..

26. ESBL (+) for *E.coli* and *Klebsiella* spp? 🞎 Yes 🞎 No

27. Was blood culture performed? 🞎 Yes 🞎 No

28. Is there any microorganism that cultured from blood culture? 🞎 Yes 🞎 No

29. Name of the microorganism : …………………………………

30. Antibiotic name that was used for urinary tract infection: ……………………………………

31.Is there any toilet room in patient’s room? 🞎 Yes 🞎 No

32.Is there any hand disinfectant in patient’s room? 🞎 Yes 🞎 No

33. How many patients are present in the room: ………….

**Section to be filled if Urinary System Catheter is present:**

34. Is the catheter necessary? 🞎 Yes 🞎 No

35. Duration of catheterization ? …………..

36. Type of Catheter: 🞎 Foley 🞎 Other *(please indicate:)* …………………...

37. Catheterization was performed at: 🞎 Emergency Room 🞎 Clinic 🞎 ICU

38. Catheterization was performed by: 🞎 Doctor 🞎 Nurse 🞎 Catheter Team 🞎 Other: …..

39. Is catheter set available? 🞎 Yes 🞎 No

40. Is routine catheter changing performed? 🞎 Yes 🞎 No

41. Is the drainage bag properly positioned? 🞎 Yes 🞎 No

42. Is the drainage bag touching the ground? 🞎 Yes 🞎 No

43. Is the level of urine drainage bag below the bed? 🞎 Yes 🞎 No

44. Has the drainage bag a tap ? 🞎 Yes 🞎 No *Thank you!*
